# Mosquito midgut *Enterobacter cloacae* and *Serratia marcescens* affect the fitness of adult female *Anopheles gambiae* s.l.

**DOI:** 10.1371/journal.pone.0238931

**Published:** 2020-09-18

**Authors:** Lilian Chiamaka Ezemuoka, Esinam Abla Akorli, Fred Aboagye-Antwi, Jewelna Akorli

**Affiliations:** 1 African Regional Postgraduate Programme in Insect Science (ARPPIS), University of Ghana, Legon, Accra, Ghana; 2 Department of Parasitology, Noguchi Memorial Institute for Medical Research, University of Ghana, Legon, Accra, Ghana; 3 Department of Animal Biology and Conservation Science, University of Ghana, Legon, Accra, Ghana; 4 West African Centre for Cell Biology of Infectious Diseases, University of Ghana, Legon, Accra, Ghana; Swedish University of Agricultural Sciences, SWEDEN

## Abstract

Some bacteria species found in the mosquito midgut have demonstrated their role in interrupting the development of *Plasmodium* within the midgut of the *Anopheles* mosquito and have been identified as potential candidates for novel bacteria-mediated disease control. However, to use these bacteria successfully in biocontrol mechanisms their effect on the fitness of the vector into which they have been introduced has to be evaluated. This study investigated the effect of two such bacteria candidates, *Enterobacter cloacae* and *Serratia marcescens*, on *Anopheles gambiae* s.l. fitness. Pupae and larvae of *Anopheles gambiae* s.l. mosquitoes were collected by dipping method and reared to adults. The effect of these bacteria on mosquito fitness was assessed by reintroducing isolates of each bacteria separately into antibiotic-treated female adult mosquitoes through sugar meal. Wild type (non-antibiotic-treated) mosquitoes and those antibiotic-treated with no bacteria reintroduction were used as controls. The mosquitoes were monitored on longevity/survival, fecundity, hatch rate, and larval survival. The antibiotic-treated adult mosquitoes had reduced life span with median survival of 14 days while the bacteria-reintroduced groups and the wild type survived to day 22 (*p*< 0.0001). Treatment with *Enterobacter* and *Serratia* did not affect the average egg deposition (*p>*0.05) but they affected hatch rates positively (*p* = 0.008). There was, however, some evidence that suggests *Enterobacter* could have a positive effect on larval development (*p* < 0.0001). With no observed negative effect on survival/longevity of *Anopheles gambiae*, introducing *E*. *cloacae* and *S*. *marcescens* in future bacteria-associated control strategies is unlikely to result in mosquitoes that will be outlived by the wild population. This, however, requires evaluations under field conditions.

## Introduction

Mosquito-borne diseases are a major concern in many parts of the world, malaria being the most critical with more than 400,000 death every year [1]. In 2018, malaria alone caused 405, 000 deaths [1]. The WHO African Region was home to 93% of malaria cases and a similarly high percentage of malaria deaths [1]. Mosquito vector control programmes have been shown to significantly reduce mosquito-borne diseases [2]. Despite control efforts, mosquito-borne diseases remain significantly high and continue to increase worldwide due to increased global travel [3] and adaptation of the vectors to new niches [4–6]. Resistance of parasites to existing drug therapies, mosquito-insecticide resistance and poor health facilities in disease endemic settings have contributed to the increasing prevalence of mosquito-borne diseases [7–9]. As vector control remains an effective method to reduce vector-borne disease prevalence, new control approaches that depend on transforming mosquitoes and/or their symbionts to increase vector refractoriness have been suggested [10].

The gut microbiota of mosquito is a heterogeneous and variable network of organisms. Several species of bacteria have been found associated with the mosquito midgut lumen using both culture-dependent and culture-independent methods [11–16]. These methods have also been used to demonstrate the acquisition of these bacteria from the mosquito larval aquatic environment [17] and trans-stadial transmission to the adult gut [18]. Microbial diversity in the mosquito midgut reduces after blood feeding with proliferation of some bacteria species [15]. The benefits of bacteria to the physiological well-being of insect vectors are varied and include nutrition, reproduction, metabolism, and immunity [19]. Gut microbiota can secrete compounds which are absorbed by mosquitoes or they may enhance digestion through the release of digestive enzymes which facilitates the absorption of nutrients [20]. *Serratia* and *Enterobacter* species, for example produce haemolytic enzymes which enhance blood digestion in blood-feeding dipterans [21]. Mosquito gut bacteria can also impact pathogen development by stimulating the host immune system [22]. The naturally acquired microflora regulate mosquito vector competence by suppressing the maturation of *Plasmodium* and other mosquito transmitted parasites [23]. An example is observed with re-introduction of an *Enterobacter* strain isolated from Zambian *Anopheles* mosquitoes (*Esp_Z*) into experimental mosquitoes which led to suppressed development of *Plasmodium* parasites and disruption of the midgut epithelium invasion by exerting oxidative pressure [24]. ‘Sterile’ mosquitoes are more susceptible to *Plasmodium* infection while co-infection with gametocytes and bacteria results in low levels of infection [14, 22]. It remains unclear whether the increased susceptibility results from weakened immunity due to the absence of the bacteria or a lack of bacteria-secreted anti-parasitic molecules.

There is increasing interest in investigating symbionts of malaria vectors because their anti-parasitic mechanisms may offer novel control techniques. To use bacteria-mediated control strategies effectively requires comprehensive understanding of how naturally occurring candidate bacteria species affect elements of vector capacity. In this study, we isolated *Enterobacter cloacae* and *Serratia marcescens*, two bacterial species which have potential to be used for novel bacteria-mediated control, from female *Anopheles gambiae* s.l. and determined their effect on the mosquito fitness by assessing longevity, fecundity, fertility and larval survival.

## Methods

### Mosquito collection and maintenance

The *Anopheles* mosquitoes, from which the bacteria used in this study were isolated, were collected from dugouts and water puddles on an urban agricultural site in Accra, Ghana (Latitude 5° 35′54.54″N and Longitude 0°10′53.60″E). Pupae and larvae of *Anopheles* mosquitoes were sourced from mosquito breeding ponds and returned to the laboratory in samples of water from the breeding ponds. In the laboratory, mosquito samples were emptied into larval trays. Pupae were transferred into plastic cups and kept in labelled cages cleaned with 70% ethanol. Remaining larvae were reared without adding fish meal, and pupae were picked and transferred into cages daily. All remaining larvae were discarded 5 days after the field collection. The rearing temperature conditions for larvae and adults were 36 ± 1° C and 25 ± 1° C respectively. All samples were maintained at 78 ± 2% relative humidity, and 12:12 (dark:light) photoperiod. Cotton balls containing 10% sugar solution were placed in the cages after taking out 20 “unfed” 1-day old adult female mosquitoes for dissection. Similarly, 20 female mosquitoes were dissected after sugar feeding for 3 days, and another 20 mosquitoes 24 hours after being blood fed. *Anopheles* mosquitoes were identified based on their morphology to species level with taxonomic keys [25].

### Ethics statement

The vegetable farm owners gave verbal permission to sample mosquitoes from their farms.

### Mosquito dissection

Dissections were performed with the aid of a *Leica* stereomicroscope (EZ4 HD) and with sterilized apparatus according to a procedure depicted by Pidiyar *et al*. [26] with slight modifications. Before dissection, female mosquitoes were aspirated from the cage into paper cups and placed on ice to immobilize them. They were washed with 75% ethanol for 5 min and then four times in sterile 1X Phosphate Buffered Saline (PBS) to wash out nonattached bacteria, thereby lessening the contamination of sample with cuticle bacteria during mosquito dissection. Each mosquito was dissected in a drop of filtered 1X PBS on a microscope slide wiped with 0.5% bleach and 70% ethanol. The tips of dissecting pins and forceps were cleaned with 0.5% bleach, absolute ethanol and 70% ethanol between each dissection to prevent contamination between sample midguts [27]. The midgut sections were dropped singly in 100μL of 1X PBS. Sham dissections (drop of PBS on a cleaned slide with no mosquito) were prepared as negative controls.

### Bacteria isolation and identification

Three replicates, each containing five midguts were prepared for unfed, sugar fed and blood fed mosquitoes. The guts were homogenized in 1X PBS and diluted serially (10 folds) up to 10^−2^. 100μL of each dilution was pour-plated on MacConkey agar, blood agar mixed with 5% sheep blood, *tryptone soya agar* (*TSA*) and *with 10% sodium chloride (NaCl)*. *The latter was* incubated at 27°C and remaining agar plates at 37°C for 24–48 hrs. An empty plate and another with sterile 1X PBS were prepared in two replicates as negative controls. Continuous subcultures of bacteria colonies were carried out to isolate pure bacteria colonies. The single pure colonies of bacteria were identified using Matrix-assisted laser desorption and ionization time-of-flight mass spectrometry (MALDI-TOF-MS). Bacteria identification at the species level was considered to be accurate and significant when the spectrum in question had a log score value (LSV) ≥ 1.9 [28]. The bacteria identities were also confirmed by PCR amplification of ~1500bp region of the bacterial small subunit ribosomal ribonucleic acid (SSU rRNA) using universal bacteria primers, sequencing and performing a Megablast search in Geneious v11.0.5 (S1 Table). Sequences of the isolate have been deposited in NCBI (Genbank submission: SUB7865016). Only one isolate each of *Enterobacter cloacae* (Mu2b) and *Serratia marcescens* (Tu2bii) were used in subsequent experiments.

### Mosquito antibiotic treatment and bacteria reintroduction

*Anopheles gambiae* s.l. mosquitoes aged 2–3 days were placed into twelve rearing cages; each cage containing 80 females and 80 males. Nine mosquito cages were maintained on cotton balls soaked in a mixture of antibiotic cocktail (75μg/mL gentamicin, 100 units/mL Penicillin and 100μg/mL of streptomycin in 10% sugar solution) for 4 days [24]. The soaked cotton balls were changed daily to prevent bacterial contamination. Three cages were maintained on a sterile 10% sugar solution only and were used as positive controls (wild type). The treated mosquitoes were allowed to feed for 24 hours on sterile 10% sugar solution after antibiotic treatment to reduce the effect of any residual antibiotics. The efficiency of bacteria clearing was tested by dissecting the midguts of 5 mosquitoes from each of the treated cages, and culturing their gut homogenate on MacConkey agar, tryptone soya agar and blood agar, as previously described. No growth was recorded. Three (3) antibiotic-treated cages each were used for *Enterobacter cloacae* and *Serratia marcescens* reintroduction. The remaining three (3) were kept without bacteria reintroduction.

An isolate of *E*. *cloacae* and *S*. *marcescens* were grown separately in nutrient broth for 24 hours at 37°C. Each culture was centrifuged for 10min at 3000rpm to obtain pellets. The supernatant was discarded and pellets were diluted in 3% sugar solution to a final OD_600nm_ of 3 [29]. Mosquitoes were starved for 6 hours before feeding with bacteria-sugar solution. Cotton balls were soaked with the bacteria-spiked sugar solution and placed in mosquito cages. Cotton balls were changed daily, and bacteria-sugar solutions were prepared fresh to use. Bacteria reintroduction was done for 48 hours.

To confirm that the introduced bacteria colonized the mosquito gut, five adult female mosquitoes were removed from each cage and dissected. The midguts were homogenized in 100μL of 1X PBS, diluted serially to 10^−4^ and plated on TSA and MacConkey agar. Plates were incubated for 24 hours at 37°C, and bacteria colonies were identified using the MALDI-TOF-MS as previously described. All treatment groups were blood-fed 4 days after bacteria reintroduction. Mosquitoes that did not feed were removed from the cages.

### Adult longevity assay

Three (3) independent replicates of 60 female and 60 male antibiotic-treated mosquitoes per cage were set-up to investigate the impact of each of the two bacteria on mosquito lifespan *E*. *cloacae* and *S*. *marcescens* were reintroduced separately. Two control groups were set-up as previously described. All cages were provided with a blood meal 4 days post bacteria reintroduction, and non-fed mosquitoes were removed from the cages. Mosquitoes were maintained on cotton balls soaked with 10% sugar solution and blood-fed twice a week until all the mosquitoes died. Female mosquitoes that did not feed were not removed from the cages. Dead female mosquitoes were removed from the cages and counted (S2 Table). Survival percentages were calculated across three biological replicates for each treatment group.

### Fecundity and fertility assay

Oviposition cups (filter paper submerged in water holding cups) were placed inside the mosquito cages used for survival assay 24 hours after blood feeding. Eggs laid on the filter paper were collected after 3–4 days and counted [24]. The mean number of eggs was calculated as the total number of eggs laid fractioned by the number of female mosquitoes in the cage. This was repeated after the 2nd blood meal (S3 Table).

Egg viability was determined by submerging the eggs laid by mosquitoes into plastic trays with dechlorinated water and allowed to hatch under standard larval rearing conditions. Hatch rate was assessed by counting first instar larvae (S4 Table).

### Larval survival assay

The 1^st^ instar larvae from the fertility assay above were maintained under standard rearing conditions to assess their developmental success. The larvae were fed daily on Tetrafin^®^ fish meal (food). The number of larvae at each developmental stage was counted and recorded daily. Pupae were removed and placed in adult cages to emerge. The number of adults that emerged was recorded (S5 Table).

### Data analysis

Data collected were analyzed using GraphPad Prism 7.04 software. Kaplan–Meier survival analysis was performed, and *p*-values defined by log-rank test (Mantel-Cox). Sidak method was used to correct for multiple comparisons. Significance for fecundity, hatch rate and percentage larval development was determined using Analysis of Variance (ANOVA).

## Results

### Bacteria species identification

All 14 bacterial samples were submitted to identification by MADI-TOF-MS. Two bacterial species were predominant among the bacteria isolated, *Enterobacter cloacae* and *Serratia marcescens* with LSV ≥ 1.9 (Table 1). *S*. *marcescens* represented 57% (8) of the 14 isolates, while 28.6% (4) were identified as *E*. *cloacae*. Two (2) samples were not identified with MALDI-TOF but 16S sequence blast search for these isolates showed >99% identity with *E*. *cloacae* complex and *Serratia* sp (Table 1 and S1 Table). An average length of 1344bp of bacterial 16S ribosomal subunit sequence was obtained for all the isolates. There was generally good sequence agreement (length and percentage identity) between our sequences and those retrieved as ‘hits’ from Genbank (Table 1 and S1 Table). The two bacteria identification results generally matched with significant LSV (≥ 1.9) and sequence identity.

**Table 1:** A summary of Bruker MALDI Biotyper and 16SrDNA identification results. MALDI-TOF bacteria identity was significant if LSV ≥ 1.9.

	MALDI-TOF		16SrDNA sequencing		
Bacteria identification number	Organism best match	Log score value (LSV)	Query sequence length	Hit sequence length	%identity with MALDI-TOF best match organism
Mu1bi	No identification possible	1.54	1430	1425	99.3^✢^
Mu1bii	*Enterobacter cloacae*	2.14	1434	1428	97.4
Mu2b	*Enterobacter cloacae*	1.91	1083	1079	98.2
Ms1a	*Enterobacter cloacae*	2.11	1460	1369	99.1
Ms1b	*Enterobacter cloacae*	1.99	1430	1430	99.8
Mb1a	No identification possible	1.65	1430	1430	99.7^✤^
Mb1b	*Serratia marcescens*	2.02	1429	1426	99.9
Bu1b	*Serratia marcescens*	2.04	1430	1430	99.8
Bs2aii	*Serratia marcescens*	2.11	1414	1409	99.7
Bb1b	*Serratia marcescens*	1.9	1433	1426	99.2
Tu2bi	*Serratia marcescens*	2.03	1429	1429	99.7
Tu2bii	*Serratia marcescens*	2.03	1418	1416	99.4
TNu1b	*Serratia marcescens*	2.21	1429	1426	99.7
TNs1b	*Serratia marcescens*	2.06	570	568	98.2
BTS (+ control)	*Escherichia coli*	2.35			
NEG C (standard)	no peaks found	0			

**Agar plates**: M stands for MacConkey agar, B stands for blood agar, T stands for TSA, TN stands for TSA + 10% NaCl.

**Midgut:** u stands for unfed, s stands for sugar fed, b stands for blood fed.

^✢^ Highest hit organism was *Enterobacter hormaechei*, a member of the *E*. *cloacae* complex.

^✤^ Similarly identical to *Serratia surfactantfaciens* and *S*. *marcescens*.

#### Effect of *Enterobacter cloacae* and *Serratia marcescens* on the longevity of *Anopheles gambiae* s.l

**Fig 1:**
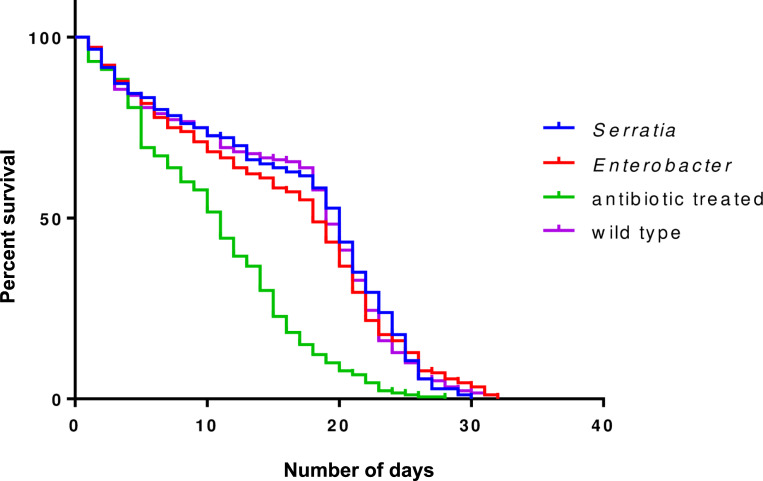
Survival analysis of *An*. *gambiae* s.l. following antibiotic treatment and bacteria reintroduction. The group, antibiotic-treated are antibiotic-treated with no bacteria reintroduction and wild type are non-antibiotic-treated.

Antibiotic-treated mosquitoes with no bacteria reintroduced had reduced lifespan compared to the wildtype, *Enterobacter-*fed and *Serratia*-fed group (*p*< 0.0001). Approximately, 50% of antibiotic-treated mosquitoes died by the 14^th^ day post blood feeding, with the rate of mortality increasing steeply. By day 20 only 15% of mosquitoes were still alive (Fig 1). The survival rate of *Enterobacter-*fed and *Serratia*-fed mosquitoes was comparable to the wildtype (*Enterobacter* vs wildtype: *p* = 0.83; *Serratia* vs wildtype: *p* = 0.70) and there was no difference between the bacteria reintroduced groups (*Enterobacter* vs *Serratia*: *p* = 0.83). Among these, 50% of mosquitoes remained 18–20 days after blood meal (Fig 1).

#### Effect of *E*. *cloacae* and *S*. *marcescens* on the fecundity and fertility of *Anopheles gambiae* s.l

**Fig 2:**
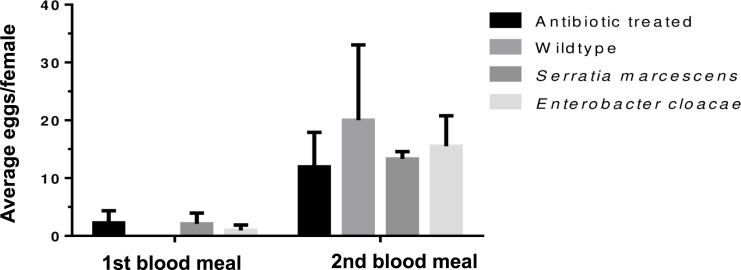
Mean number of eggs laid by *Anopheles gambiae* s.l. following antibiotic treatment and reintroduction of *E*. *cloacae* and *S*. *marcescens*. Error bars show the standard error of mean.

**Fig 3:**
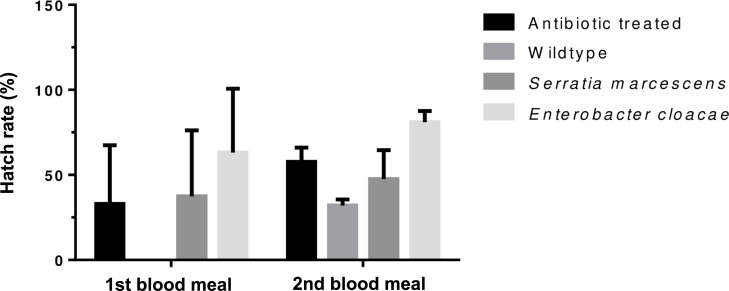
Average percentage hatch rate of *An*. *gambiae s*. *l*. eggs after antibiotic treatment and bacteria reintroduction of *E*. *cloacae* and *S*. *marcescens*. Error bars show the standard error of mean.

Eggs laid by experimental group of mosquitoes were collected on filter papers, counted and hatched. *Enterobacte*r-fed, *Serratia*-fed and the antibiotic-treated groups laid eggs after each blood meal while the wild type laid eggs only after the second blood meal (S3 Table). The average number of eggs laid was similar between treatment after each blood meal (*p =* 0.81) (Fig 2) but hatch rates differed significantly (*p =* 0.008) (Fig 3). The mean number of eggs (*p* = 0.0001) and hatch rate increased following the second blood meal (*p* = 0.04).

#### Effect of *Enterobacter cloacae* and *Serratia marcescens* on survival of larval *Anopheles gambiae* s.l

**Fig 4:**
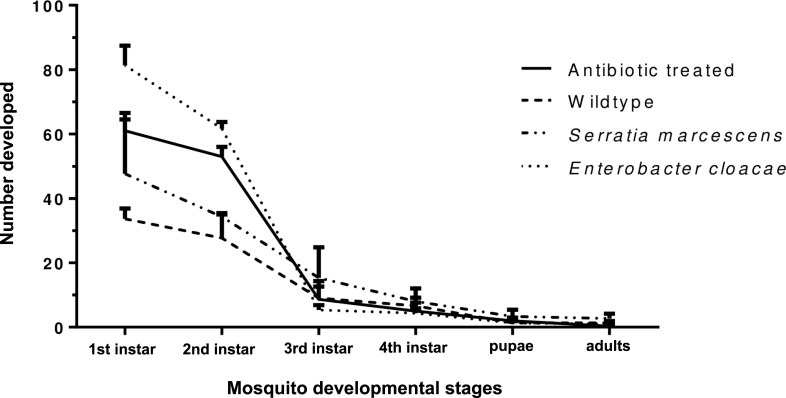
Developmental successes of *An*. *gambiae* from eggs to adults following bacteria reintroduction. Percentages were calculated based on the number of eggs laid.

We observed the effect of *Enterobacter* and *Serratia* on mosquito survival from eggs to adults (Fig 4). There was evidence of significant effect of treatment on the percentage number of mosquitoes that reached 2nd instar larvae (*p<*0.0001). The developmental successes were however similar between groups from 3^rd^ instar to adults. *Enterobacter***-**fed group had the highest percentage of 1^st^ and 2^nd^ instar larvae and these differed from those observed in other treatments, except with 2^nd^ instars from antibiotic-treated adult mosquitoes (S5 Table).

## Discussion

*Enterobacter cloacae* and *Serratia marcescens* are two bacteria species within the midgut of mosquitoes that have shown promising results in reducing parasite development in the vector host [22–24]. Their use as potential bio-agents for control of disease transmission from mosquito to human host has been of scientific interest. In this study, we investigated the effects of *E*. *cloacae* and *S*. *marcescens* on adult *An*. *gambiae* s.l survival and fertility and eggs to adult developmental success. The effect of two bacteria species were compared with antibiotic-treated’ mosquitoes and wildtype (non antibiotic-treated). Our results show evidence that *E*. *cloacae* and *S*. *marcescens* may contribute to mosquito survival fertility and fecundity.

Microorganisms resident in the midgut of insects are important in many physiological processes in the host [30]. The bacteria family Enterobacteriacae, of which *E*. *cloacae* and *S*. *marcescens* belong, show increased abundance in the mosquito midgut following a blood meal [31, 32]. They may be playing immune functions [22], digesting food [21] to support the mosquitoes physiological well-being. Antibiotic treatment of mosquitoes is not efficient to remove all bacteria within the midgut [31]. It, however, allows specific bacteria of interest to be studied for their physiological effects by reintroduction into antibiotic-treated mosquitoes. We have demonstrated the importance of *E*. *cloacae* and *S*. *marcescens* to the survival of adult mosquitoes. The reduced longevity in antibiotic-treated mosquitoes may have resulted from reduced bacterial abundance [14, 33] or the removal of some specific bacteria which have significant roles in keeping the mosquitoes healthy. The absence of the two bacteria of interest was confirmed, after antibiotic treatment. Therefore, the rescue of longevity after reintroduction of *Enterobacter* and *Serratia* into antibiotic-treated mosquitoes demonstrated that the two bacteria species were independently important in the general survival of adult *Anopheles gambiae* s.l. mosquitoes. This is in agreement with similar longevity studies with *E*. *cloacae* strain (*Esp_Z*) isolated from *Anopheles* mosquito population [24, 34].

We observed no difference in fecundity for *Enterobacter* or *Serratia-*treated mosquitoes compared to antibiotic-treated or the wildtype, but they differed significantly compared in fertility compared to the wildtype. There is however evidence to suggest that removal of Enterobacteriacae in mosquitoes by addition of antibiotics to blood meal have positive effect on fecundity and fertility [31]. This supports our results because the presence of either of the two bacteria species prior to blood feeding did not offer any fecundity advantage and mosquitoes laid similarly to the antibiotic-treated. *Enterobacter-*treated mosquitoes show some evidence of increased ability of their eggs surviving better to the 2^nd^ instar, but this advantage disappeared in the subsequent developmental stages.

## Conclusions

*Enterobacter cloacae* and *Serratia marcescens* are candidates for use in potential *Plasmodium* blocking strategies. The implication of our findings to this suggests that increase of abundance of the two bacteria species in the midgut of *Anopheles* mosquitoes [29] does not confer a fitness disadvantage.

## Supporting information

S1 Table BLAST search results for 14 bacterial isolates. Consensus sequences were built with partial forward and reverse sequences of bacterial 16SrRNA and searched using Megablast in Geneious v 11.0.5.
(XLSX)

S2 Table Mortality data. The number of mosquitoes that died daily was recorded for the treatment and control.
(DOCX)

S3 Table Fecundity data. The mean number of eggs was calculated as the total number of eggs laid fractioned by the number of female mosquitoes in the cage.
(DOCX)

S4 Table Fertility/hatch rate. The number of emerged 1^st^ instar larvae was recorded.
(DOCX)

S5 Table Percentage larval development. Larval development was monitored to adult and recorded.
(DOCX)

## Abbreviations

LSV: log score value
MALDI-TOF-MS: Matrix-assisted laser desorption and ionization time-of-flight mass spectrometry
TSA: *Tryptone soya agar*
