# Supplementary material for: Mosquito midgut Enterobacter cloacae and Serratia marcescens affect the fitness of adult female Anopheles gambiae s.l
Source: PLoS One. 2020 Sep 18;15(9):e0238931. doi: 10.1371/journal.pone.0238931 (PMC7500640; doi:10.1371/journal.pone.0238931)
Supplement: S2 Table — The number of mosquitoes that died daily was recorded for the treatment and control. (DOCX) [file pone.0238931.s002.docx]

**S2 Table, MORTALITY DATA**

| day | *Serratia-fed* | *Enterobacter-fed* | Antibiotic- treated | wild type |
| --- | --- | --- | --- | --- |
|  |  |  |  |  |
| 1 | 6 | 5 | 12 | 5 |
| 2 | 9 | 9 | 4 | 9 |
| 3 | 8 | 8 | 5 | 11 |
| 4 | 5 | 6 | 14 | 3 |
| 5 | 2 | 5 | 20 | 6 |
| 6 | 6 | 7 | 4 | 3 |
| 7 | 3 | 5 | 6 | 3 |
| 8 | 4 | 2 | 7 | 1 |
| 9 | 2 | 5 | 4 | 3 |
| 10 | 4 | 5 | 11 | 4 |
| 11 | 1 | 3 | 13 | 6 |
| 12 | 4 | 5 | 9 | 2 |
| 13 | 7 | 3 | 5 | 1 |
| 14 | 2 | 2 | 12 | 2 |
| 15 | 2 | 5 | 13 | 1 |
| 16 | 2 | 2 | 8 | 1 |
| 17 | 2 | 4 | 6 | 3 |
| 18 | 6 | 11 | 5 | 11 |
| 19 | 10 | 10 | 4 | 17 |
| 20 | 17 | 12 | 4 | 13 |
| 21 | 15 | 13 | 2 | 15 |
| 22 | 10 | 14 | 4 | 15 |
| 23 | 10 | 7 | 4 | 15 |
| 24 | 11 | 3 | 1 | 6 |
| 25 | 13 | 6 | 1 | 5 |
| 26 | 9 | 9 | 1 | 8 |
| 27 | 5 | 1 | 0 | 1 |
| 28 | 0 | 3 | 1 | 3 |
| 29 | 3 | 2 | 0 | 2 |
| 30 | 2 | 2 | 0 | 1 |
| 31 | 0 | 3 | 0 | 1 |
| 32 | 0 | 3 | 0 | 3 |
