# Supplementary material for: Mosquito midgut Enterobacter cloacae and Serratia marcescens affect the fitness of adult female Anopheles gambiae s.l
Source: PLoS One. 2020 Sep 18;15(9):e0238931. doi: 10.1371/journal.pone.0238931 (PMC7500640; doi:10.1371/journal.pone.0238931)
Supplement: S3 Table — The mean number of eggs was calculated as the total number of eggs laid fractioned by the number of female mosquitoes in the cage. (DOCX) [file pone.0238931.s003.docx]

**FECUNDITY DATA**

|  | Aseptic | | | Wildtype | | | *Serratia* | | | *Enterobacter* | | |
| --- | --- | --- | --- | --- | --- | --- | --- | --- | --- | --- | --- | --- |
| Ist blood meal | 0 | 4.2 | 2.5 | 0 | 0 | 0 | 3.57 | 0 | 2.7 | 1.86 | 0 | 1 |
| 2nd blood meal | 6.8 | 18.5 | 10.5 | 6.8 | 32.8 | 20.5 | 14.7 | 12.3 | 13 | 10.2 | 20.8 | 15.5 |
|  |  |  |  |  |  |  |  |  |  |  |  |  |
