# Supplementary material for: Mosquito midgut Enterobacter cloacae and Serratia marcescens affect the fitness of adult female Anopheles gambiae s.l
Source: PLoS One. 2020 Sep 18;15(9):e0238931. doi: 10.1371/journal.pone.0238931 (PMC7500640; doi:10.1371/journal.pone.0238931)
Supplement: S4 Table — The number of emerged 1st instar larvae was recorded. (DOCX) [file pone.0238931.s004.docx]

**FERTILITY/HATCH RATE DATA**

|  | Aseptic | | | Wildtype | | | *Serratia* | | | *Enterobacter* | | |
| --- | --- | --- | --- | --- | --- | --- | --- | --- | --- | --- | --- | --- |
| 1st blood meal | 0 | 68.8 | 34.2 | 0 | 0 | 0 | 77.3 | 0 | 30.5 | 76.4 | 92.3 | 80 |
| 2nd blood meal | 56.1 | 66.7 | 50.2 | 36.1 | 29.6 | 30.3 | 67 | 35.6 | 40 | 75.9 | 88.3 | 79 |
