# Supplementary material for: Mosquito midgut Enterobacter cloacae and Serratia marcescens affect the fitness of adult female Anopheles gambiae s.l
Source: PLoS One. 2020 Sep 18;15(9):e0238931. doi: 10.1371/journal.pone.0238931 (PMC7500640; doi:10.1371/journal.pone.0238931)
Supplement: S5 Table — Larval development was monitored to adult and recorded. (DOCX) [file pone.0238931.s005.docx]

PERCENTAGE LARVAL DEVELOPMENT

|  | Aseptic | | | Wildtype | | | *Serratia* | | | *Enterobacter* | | |
| --- | --- | --- | --- | --- | --- | --- | --- | --- | --- | --- | --- | --- |
| 1st instr | 56 | 67 | 60 | 36 | 30 | 35 | 67 | 36 | 40 | 76 | 88 | 80 |
| 2nd instr | 56 | 53 | 50 | 36 | 22 | 25 | 35 | 33 | 35 | 61 | 64 | 60 |
| 3rd instr | 15 | 4 | 7 | 12 | 5 | 10 | 26 | 8 | 12 | 4 | 7 | 5 |
| 4th instr | 8 | 3 | 4 | 9 | 4 | 7 | 12 | 4 | 8 | 3 | 6 | 4 |
| Pupae | 3 | 1 | 2 | 2 | 1 | 1 | 5 | 1 | 4 | 2 | 1 | 1 |
| Adult | 1 | 0 | 0 | 2 | 1 | 1 | 4 | 1 | 3 | 1 | 1 | 1 |
|  |  |  |  |  |  |  |  |  |  |  |  |  |
